# Osteitis Fibrosa Cystica

**DOI:** 10.1002/jbm4.10403

**Published:** 2020-09-07

**Authors:** Waldemar Misiorowski, John P Bilezikian

**Affiliations:** ^1^ Endocrinology Department, Centre of Postgraduate Medical Education Bielanski Hospital Warsaw Poland; ^2^ Department of Medicine, Endocrinology Division, College of Physicians and Surgeons Columbia University New York 10032 NY USA

**Keywords:** CELL/TISSUE SIGNALING ‐ ENDOCRINE PATHWAYS, PTH/VIT D/FGF23, DISORDERS OF CALCIUM/PHOSPHATE METABOLISM, PARATHYROID‐RELATED DISORDERS, RADIOLOGY

## Abstract

Osteitis fibrosa cystica is a rare presentation of both primary and secondary hyperparathyroidism. In this perspective, we provide a historical backdrop to this form of parathyroid disease and contend that this clinical presentation of excess parathyroid hormone, particularly in primary hyperparathyroidism, is still seen today. In view of its rarity and the way it typically presents, the diagnosis of metastatic cancer is often the first diagnostic impression. © 2020 The Authors. *JBMR Plus* published by Wiley Periodicals LLC on behalf of American Society for Bone and Mineral Research.

In a recent issue of the *New England Journal of Medicine*, Ramon and Berthod presented a case of extensive osteitis fibrosa cystica (OFC) caused by the secondary hyperparathyroidism of chronic, uncontrolled renal failure.^(^ ^1^ ^)^ Such cases now are rare because most patients with end‐stage renal disease are managed well enough to prevent this outcome. The case reflects a time when OFC was a common, if not classic, manifestation of renal osteodystrophy. Now, however, proactive therapeutic management has led to adynamic bone disease as a relatively more common manifestation of renal osteodystrophy. The challenge in the modern management of renal failure is to prevent either outcome. Nevertheless, full‐blown OFC can occur, particularly in countries or settings in which renal management has not been optimized.

The historical perspective of OFC describes it primarily as a classical skeletal manifestation of primary hyperparathyroidism (PHPT), not secondary hyperparathyroidism. It was first described by von Recklinghausen in 1891.^(^ ^2^ ^)^ This historical context might extend even earlier because von Recklinghausen referred to a description of a case by Engel in 1864.^(^ ^3^ ^)^ Von Recklinghausen, actually, did not suspect a relationship of OFC with a disease of the parathyroid glands, which had only been defined as an anatomical entity a decade or so before von Recklinghausen's article. The putative association between OFC and a parathyroid tumor was first recorded in 1904 by Askanazy.^(^ ^4^ ^)^ It was 2 years later that the interrelationshipbetween the parathyroid glands and calcium metabolism was discovered. It took another two decades for a parathyroid tumor to be removed from a patient with OFC in 1925.

In Fuller Albright's 1948 review of the history of PHPT, he accepted only 1 of the 3 patients described by von Recklinghausen as a true case of OFC. He attributed the other 2 to polyostotic fibrous dysplasia.^(^ ^5^ ^)^ Nevertheless, this early experience established OFC as a classic feature of PHPT, and a key aspect of its presentation through the 1960s. Then, rather quickly in the 1970s and thereafter, automated methods for measuring blood calcium levels changed dramatically the clinical profile of PHPT from a symptomatic disease to a relatively asymptomatic one. OFC was no longer a regular feature of the disease.

OFC is characterized clinically by bone pain and radiographically by subperiosteal bone resorption, osteolysis of the distal clavicles, a “salt and pepper” appearance of the skull, bone cysts, and brown tumors of the bones.^(^ ^6^ ^)^ This classical skeletal involvement still can be found. We recently described four cases of PHPT that shared an interesting common history (Fig. 1). The first clinical manifestation of the disease was a pathological fracture that masqueraded as a malignancy.^(^ ^7^ ^)^ The presence of large osteolytic lesions gave rise to the initial diagnosis of a primary or metastatic cancer. In none of the reported cases was PHPT with OFC considered as the diagnosis. One might reasonably ask how could the “obvious” radiological appearance of OFC be missed? It would seem that this question could best be explained by the fact that in many countries the incidence of OFC among patients with PHPT is so rare that in a majority of medical centers, routine skeletal X‐rays are not undertaken or even recommended.^(^ ^7^ ^)^ Even when X‐rays are taken in someone with hypercalcemia and symptoms, multiple bony lesions representing brown tumors are often misdiagnosed as metastatic carcinoma, bone cysts, osteosarcoma, or giant‐cell tumors. Because these radiological features (eg, cyst‐like radiolucency) overlap and can be characteristic of these other diseases, the diagnosis can be difficult.^(^ ^8^, ^9^, ^10^, ^11^, ^12^ ^)^ Even bone scintigraphy, which will show “hot spots” and/or a generally high uptake in PHPT, lacks specificity because it can also be appreciated in a variety of other high bone turnover conditions, such as trauma, infections, malignancy, osteomalacia, Paget disease, and other metabolic bone diseases. In particular, the metastases of cancer are also characterized by multiple focal lesions like PHPT. Even histology cannot guarantee a correct diagnosis: Brown tumors, giant cell tumors, granulomas, aneurysmal bone cysts, and some osteosarcomas can show similar PHPT macroscopic and microscopic features.^(^ ^13^, ^14^, ^15^, ^16^, ^17^ ^)^ Moreover, if hypercalcemia is present, the first impression is often that of a malignancy itself. PET scanning does not reliably distinguish between a skeletal malignancy and benign disease.^(^ ^18^ ^)^

**Fig 1:**
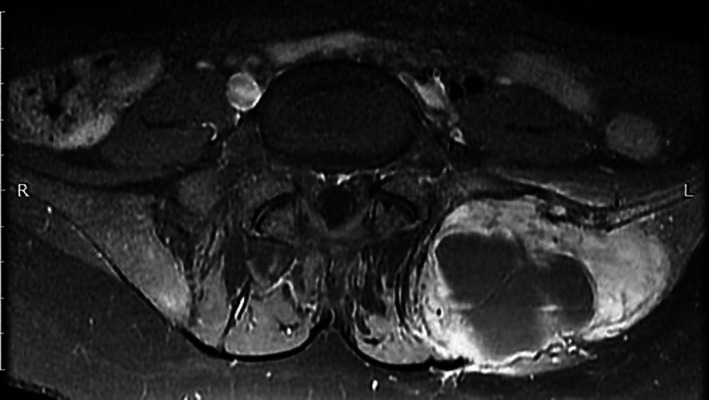
MRI showing a brown tumor of the left iliac bone of a 24‐year‐old woman.

Overt bone disease could reflect a delay in detecting PHPT in countries where routine biochemical screening is not practiced, or it could be a manifestation of excess PTH action in the face of marginal or deficient vitamin D and calcium intake. In routine biochemical screening, it has been shown that when such practices become more common, the clinical presentation of PHPT becomes less severe.^(^ ^19^ ^)^ In some places of the world, where biochemical screening is not routine (eg, India), OFC can be readily detected in PHPT. Vitamin D deficiency looms also as an important element in those countries where biochemical screening is not routine. Many studies have now confirmed that manifestations of PHPT are worse when vitamin D deficiency is present. For example, the renal and skeletal manifestations of PHPT were much worse in a Chinese cohort in which the average 25‐hydroxyvitamin D concentration was 8.8 ng/mL, than in a US cohort in which vitamin D deficiency was much less evident.^(^ ^20^ ^)^ Even in mild PHPT, Walker and colleagues have shown that the biochemical and histomorphometric manifestations of PHPT are worse.^(^ ^21^ ^)^ Whereas in centers where metabolic bone diseases are featured and evaluations tend to be much more extensive, another form of PHPT has been recognized in which the total and ionized serum calcium concentrations are normal.^(^ ^22^ ^)^ The clinical presentations of PHPT, therefore, vary according to the disposition of a country's health care providers to routinely measure serum calcium and/or to be proactive in the evaluation of metabolic bone diseases, and whether the country's population tends to suffer from vitamin D deficiency.^(^ ^23^, ^24^ ^)^

Nevertheless, a worthy question is whether the absolute incidence of severe forms of PHPT with classic, overt skeletal involvement (eg, OFC), has indeed significantly decreased, or whether these forms escape attention because the growing number of less‐severe forms of the disease predominate. Stated in another way, it is possible that the absolute incidence of severe PHPT with OFC has not changed, but its prevalence among a much greater universe of asymptomatic patients has made it seem so.

## Disclosures

The authors have no conflicts to declare.

## Author Contributions

**Waldemar Misiorowski:** Conceptualization; data curation; methodology; writing‐original draft; writing‐review and editing. **John P. Bilezikian:** Conceptualization; project administration; supervision; writing‐review and editing.

### Peer Review

The peer review history for this article is available at https://publons.com/publon/10.1002/jbm4.10403.
